# Keratin 13 expression reprograms bone and brain metastases of human prostate cancer cells

**DOI:** 10.18632/oncotarget.13175

**Published:** 2016-11-07

**Authors:** Qinlong Li, Lijuan Yin, Lawrence W. Jones, Gina C-Y Chu, Jason B-Y. Wu, Jen-Ming Huang, Quanlin Li, Sungyong You, Jayoung Kim, Yi-Tsung Lu, Stefan Mrdenovic, Ruoxiang Wang, Michael R. Freeman, Isla Garraway, Michael S. Lewis, Leland W. K. Chung, Haiyen E. Zhau

**Affiliations:** ^1^ Uro-Oncology Research Program, Department of Medicine, Cedars-Sinai Medical Center, Los Angeles, CA, USA; ^2^ Urological Research, Huntington Medical Research Institutes, Huntington Memorial Hospital, Pasadena, CA, USA; ^3^ Biostatistics and Bioinformatics, Department of Medicine, Los Angeles, CA, USA; ^4^ Department of Surgery, Cedars-Sinai Medical Center, Los Angeles, CA, USA; ^5^ John H. Stroger, Jr. Hospital of Cook County, Chicago, IL, USA; ^6^ Department of Urology and Jonsson Comprehensive Cancer Center, David Geffen School of Medicine at UCLA, Los Angeles, CA, USA and Division of Urology, Greater Los Angeles Veteran's Affairs Healthcare System, Los Angeles, CA, USA; ^7^ Sepulveda Research Corporation VA Medical Center, Los Angeles, CA, USA; ^8^ Current address: Department of Pathology, Xijing Hospital, Fourth Military Medical University, Xi'an, Shaanxi, China

**Keywords:** bone and brain metastases, RANKL-independent, targeting, biomarker, cell signal network

## Abstract

Lethal progression of prostate cancer metastasis can be improved by developing animal models that recapitulate the clinical conditions. We report here that cytokeratin 13 (KRT13), an intermediate filament protein, plays a directive role in prostate cancer bone, brain, and soft tissue metastases. KRT13 expression was elevated in bone, brain, and soft tissue metastatic prostate cancer cell lines and in primary and metastatic clinical prostate, lung, and breast cancer specimens. When KRT13 expression was determined at a single cell level in primary tumor tissues of 44 prostate cancer cases, KRT13 level predicted bone metastasis and the overall survival of prostate cancer patients. Genetically enforced KRT13 expression in human prostate cancer cell lines drove metastases toward mouse bone, brain and soft tissues through a RANKL-independent mechanism, as KRT13 altered the expression of genes associated with EMT, stemness, neuroendocrine/neuromimicry, osteomimicry, development, and extracellular matrices, but not receptor activator NF-κB ligand (RANKL) signaling networks in prostate cancer cells. Our results suggest new inhibitors targeting RANKL-independent pathways should be developed for the treatment of prostate cancer bone and soft tissue metastases.

## INTRODUCTION

Keratin is a family of intermediate filament proteins expressed by epithelial cells in a cell-specific and differentiation-dependent manner. Keratin proteins (KRTs) are classified into neutral-basic and acidic types, encoded by more than 50 genes in separate clusters on chromosomes 12 and 17 [1]. KRTs form paired filamentous complexes that support the structural integrity and functions of cells and organisms. Differential expression of individual KRTs in various carcinomas make them useful biomarkers for the histopathologic diagnosis of tumors. The transcriptional regulation of distinct *KRT* genes and the functions of the encoded KRT protein filaments mediating specific structural and regulatory functions controlling tissue-specific cell growth and differentiation remain to be determined [2]. Keratin 13 (KRT13), a 54 kDa type 1 acidic intermediate filament protein often paired with KRT4, is expressed in suprabasal layers of non-cornified stratified squamous epithelia [3]. KRT13 was implicated in urothelial and stem cell differentiation [4], and has a diverse level of expression in cancer. Lower KRT13 expression, in comparison to the matching normal squamous tissues, was found in oral dysplasia, squamous carcinomas and carcinoma *in situ* [5], esophageal squamous cell carcinoma [6], bladder cancer [7], lymph node-positive uterine cervix cancer [8], and head and neck squamous cell carcinoma cell lines [9]. By contrast, higher KRT13 expression was detected in colorectal cancer [10], gastric cancer [11], and tongue squamous cell carcinoma [12]. Hamagawa, *et al.* [13], reported that despite a lower level of KRT13 protein expression in cervical cancer compared to controls, increased KRT13 mRNA nevertheless can be detected in micrometastases in the lymph nodes of cervical cancer by reverse transcription-polymerase chain reaction (RT-PCR). KRT13 expression can be induced by the activation of phosphatidylinositol 3-kinase (PI3K) in papilloma cells and induces the normal differentiation of human mucosal keratinocytes [14]. In breast cancer, a 2.5 kb upstream estrogen receptor (ER)-binding regulatory region for KRT13 was identified and three estrogen response elements and three Sp1 sites were found to be involved in its ligand-dependent differential recruitment of ER and co-activators for the induction of KRT13 expression [14]. In human and murine gastric epithelial cells, KRT13 was identified as a novel chenodeoxycholic acid-regulated farnesoid X receptor/NR1H4-target gene [11]. He et al. [15], showed that Krüppel-like factor 4 (KLF4) transcriptionally regulates KRT13 resulting in the induction of esophageal squamous cell carcinoma differentiation. A heterozygous missense mutation of mucosal KRT13 is closely associated with an inherited form of leukokeratosis or oral white sponge nevus [16]. Despite enhanced tissue-specific KRT13 protein expression in several cancer types, its potential function in different stages of cancer progression and metastasis has not been elucidated.

This communication delineates the potential functional role of KRT13 in human prostate cancer growth, development, progression, and metastasis. We examined the basal levels of KRT13 expression in developing human prostate and in three lineage-related isogenic prostate cancer bone metastatic progression cell models, and validated KRT13 expression in an aggressive and metastatic CWR22Rv1 model. Because within lineage-related prostate cell lines, KRT13 expression was consistently elevated in the aggressive isogenic cell lines, we examined the potential directive roles of KRT13 in the indolent or less aggressive prostate cancer cells to express increasingly aggressive and metastatic phenotypes. To understand its pathophysiological significance, KRT13 expression was also evaluated in clinical human primary prostate cancer tissues, prostate cancer bone metastasis, and lung and breast cancer bone and brain metastatic specimens. Differential expression of genes in KRT13-transfected prostate cancer cells confirmed the altered expression of epithelial-to-mesenchymal transition (EMT)-, stemness-, neuroendocrine-/neuromimicry-, osteomimicry-, develop- mental- and extracellular matrix-related genes. This work represents the first finding that KRT13, a structural intermediate filament protein responsible for the maintenance of the integrity of epithelial cells by attaching to the cell plasma membrane via desmosomes, could have direct regulatory functions in cancer invasion, migration, and metastasis to bone, brain, and other soft tissues.

## RESULTS

### Co-expression of KRT13 and KRT4 in developing, benign, and malignant prostate glands

**Figure 1:**
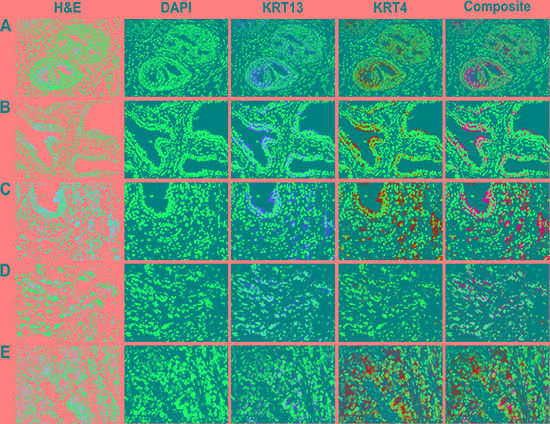
Expression of KRT13 and KRT4 in developmental, benign, and malignant prostate glands Co-expression of KRT13 and KRT4 was detected in a 4-month-old fetal (**A**), normal (**B**), and malignant prostate (**C**). The co-expression of KRT13 and KRT4 was disrupted in some other prostate cancer glands (**D** and **E**).

Because KRT13 located at the suprabasal layer of glandular epithelia and could participate in prostate development, we stained KRT13 in a 4 month-old fetal human prostate gland to confirm the expression of KRT13 in developing prostate. Figure 1A shows the parallel expression of KRT13 and KRT4 protein within the luminal epithelial- and basal cell-layers of the prostate gland. While KRT13 and KRT4 were co-expressed in normal fetal prostate gland and benign human prostate glands (Figure 1B), the co-expression of these KRTs was more variable in malignant prostate tissues, where KRT13 and KRT4 were either co-expressed or uncoupled (Figure 1C–1E).

### KRT13 expression in primary hormone-naïve prostate cancer tissues correlates with progression and overall survival of prostate cancer patients

**Figure 2:**
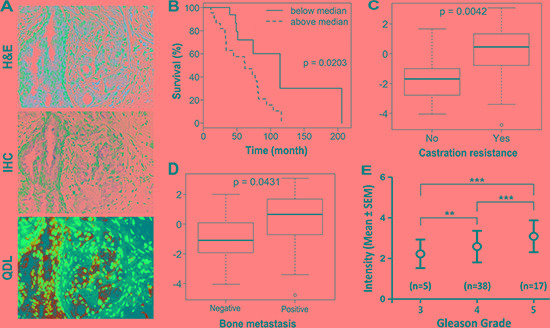
KRT13 expression in prostate cancer correlates with the progression and overall survival of prostate cancer patients (**A**) KRT13 expression detected by quantum dot labeling (QDL) is more sensitive than conventional IHC. The immunoreactivity can be quantified on a single cell basis. Analyses of QDL of KRT13 in primary prostate cancer tissue specimens from 44 hormone-naïve patients showed that KRT13 expression detected and quantified by QDL correlates significantly with patient overall survival (**B**), status of castration resistance (**C**), bone metastasis (**D**), and the Gleason grade (**E**).

Because the stemness of cancer cells correlates with cancer cell migration, invasion and drug resistance and their transition to expressing a mesenchymal phenotype [17, 18], we tested the hypothesis that KRT13 expression in clinical prostate cancer tissues correlates positively with disease progression and negatively with the overall survival of prostate cancer patients. Figure 2A shows positive staining of KRT13 in both basal and luminal prostate epithelial cell compartments but negative staining in the fibromuscular stroma compartment of the prostate cancer tissues. While lower levels of KRT13 expression were associated with the basal and luminal epithelial cell compartments of the adult normal and benign hyperplastic prostate glands, elevated KRT13 expression, in comparison, was found in invasive human prostate cancer cells. Our laboratory has established an immunohistochemical method using a quantum dot labeling (QDL) technique in which prostate cancer cell biomarker staining is more sensitive and the immunoreactivity can be quantified [19, 20]. Employing prostate cancer cell QDL in a cohort of well-defined primary prostate cancer tissue specimens collected from 44 hormone-naïve patients [21], we demonstrated that KRT13 expression, at a single cell level, correlates with the overall survival of prostate cancer patients (Figure 2B). Increased KRT13 expression also correlates significantly with castration resistance (Figure 2C), bone metastasis (Figure 2D), and Gleason grade (Figure 2E) of prostate cancer patients. Importantly, in this same set of patients we confirmed a previously reported finding from our laboratory, that the expression of an osteomimicry-related receptor activator of NF-κB ligand (RANKL), also correlated with the survival of prostate cancer patients [22].

### KRT13 expression correlates positively with prostate cancer cell proliferation, migration, and invasion in culture and in bone and brain metastases in mice: Confirmation of elevated KRT13 expression in clinical metastatic cancer specimens

**Figure 3:**
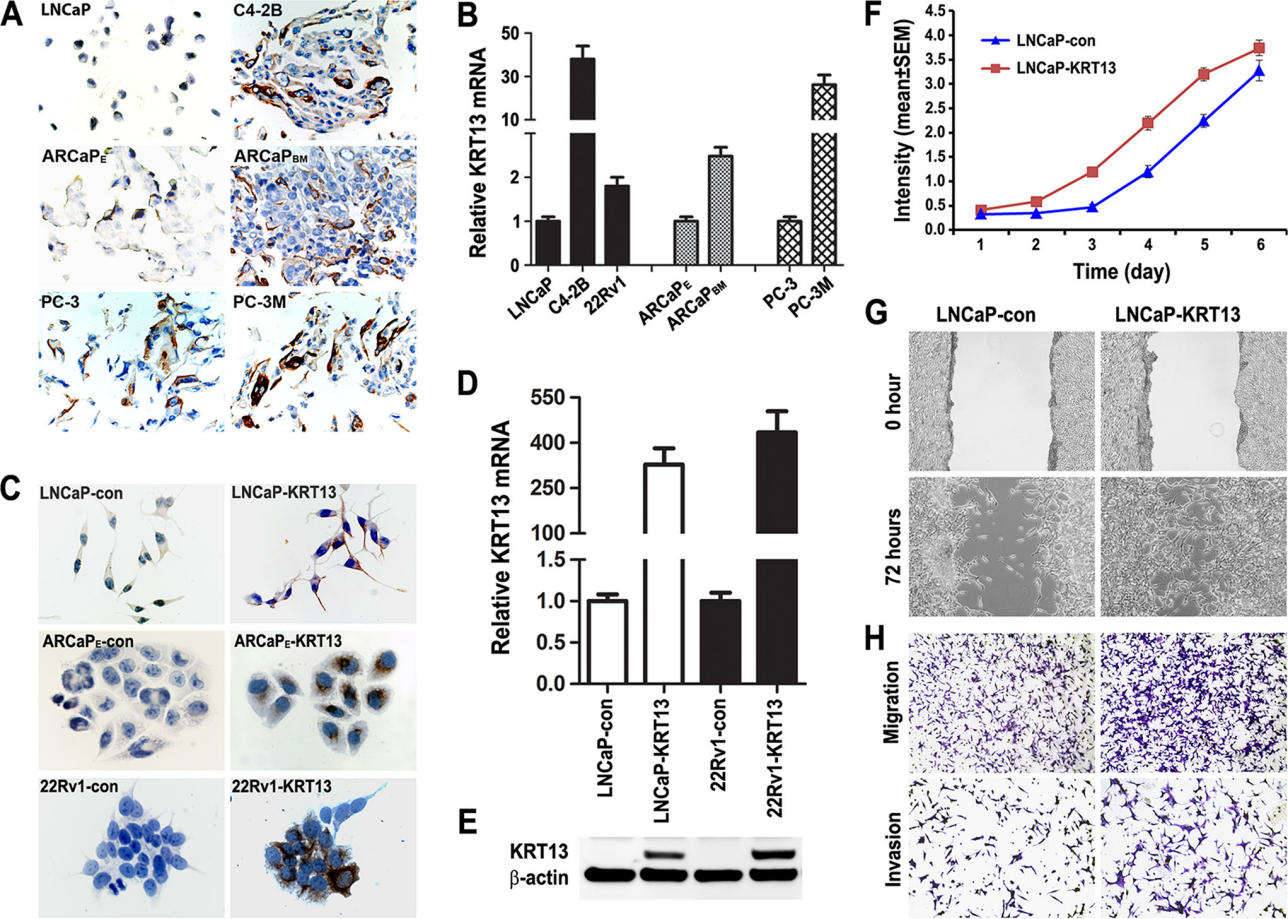
KRT13 expression correlates with cancer progression in three metastatic prostate cancer cell models (**A**) Higher levels of KRT13 proteins were detected in the metastatic C4-2B, ARCaPBM, and PC-3M cells than their respective parental cells (×500). Similar results were confirmed by qRT-PCR (**B**). KRT13-overexpressing LNCaP, ARCaPE and 22Rv1 cells were established by KRT13 gene transduction and confirmed by IHC staining (**C**), qRT-PCR (**D**), and western blot (**E**). LNCaP-KRT13 cells showed higher proliferation (**F**), migration (**G**), and invasion (**H**) phenotypes than LNCaP-neo control cells.

We examined KRT13 protein expression in three lineage-related isogenic human prostate cancer bone metastatic cell models, LNCaP/C4-2B, ARCaPE/ARCaPBM, and PC-3/PC-3M [23–25]. Figure 3A and 3B show elevated KRT13 protein and mRNA expression in all three of the bone metastatic variants in comparison to their parental control cells.

KRT13 level in prostate cancer bone metastasis appear to be cell context-dependent, since despite the ability of 22Rv1 cell model [26] to exhibit bone- and brain metastasis potential, these cells expressed low levels of KRT13 compared to their KRT-transduced counterparts as assessed by IHC (Figure 3C), qRT-PCR (Figure 3D), and western blot (Figure 3E). We generated KRT13-stably transfected LNCaP, ARCaPE, and 22Rv1 cells along with their respective neo-transfected control cells to assess their *in vitro* and *in vivo* behaviors. These cell lines are known to express androgen receptor (AR) and prostate specific antigen (PSA), two well-known prostate cancer markers.

**Figure 4:**
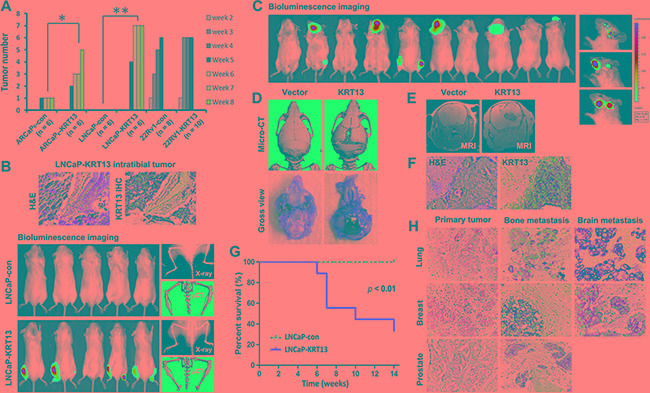
KRT13-overexpression reprograms bone and brain metastases in mice (**A**) Intratibial injection of KRT13-overexpressing LNCaP cells induced higher incidence of tumor formation. The numbers of tumors formed were counted weekly for 8 weeks in mice inoculated with ARCaPE and LNCaP cells and for 5 weeks in mice inoculated with 22Rv1 cells. (**B**) Top panel, KRT-13 expressed in bone metastatic tumors induced by LNCaP-KRT13 cells via intratibial injection. Bottom panel, bioluminescence imaging of bone tumor colonization and KRT13 expression in the tumor are shown. For each group, a representative X-ray image and micro-CT scan of the tibias were shown. (**C**) Representative bioluminescence images (week 10) of brain and bone metastases in mice with intracardiac injection of LNCaP-KRT13 cells. Representative micro-CT and gross morphology (**D**) MRI (**E**) and KRT13 expression (**F**), showing tumor growth, compression, and cracking of skull. The mice had shorter survival than those injected with LNCaP-neo (**G**). The incidences of metastatic sites and number of mice per group were: brain only (5), bone only (1), and brain and bone (4). KRT13 expression in brain or bone metastatic tissues from lung, breast, and prostate cancer tissues are higher than those in their respective primary cancer tissues (**H**).

In our study, we observed KRT13 consistently increased cell proliferation, migration and invasion of all three prostate cancer cell lines, but these cells expressed variable levels of genes associated with EMT, stemness and neuroendocrine (NE) phenotype (see below and Supplementary Figure S1A and Figure S1B). As an example, KRT13 overexpression in LNCaP cells enhanced cell proliferation (Figure 3F), migration assessed by either cell scratch assay or by transwell assay (without Matrigel-coating) and invasion (with Matrigel coating) (Figure 3G and 3H). Corresponding to the increased cell proliferation, migration and invasion, KRT13 greatly increased the total number of tumors detected in mice compared to the controls when cells were injected intratibially (Figure 4A). Here we showed that bone metastases were detected by bioluminescence imaging, histopathology, and immunohistochemistry (Figure 4B). When KRT13 overexpressing cells were injected intracardially, metastatic tumors were detected largely in bone and brain. as confirmed by bioluminescence imaging, tumor histopathology and MRI/micro-CT imaging with the latter showed disrupted and cracking skulls in mice (Figure 4C–4F). As expected, the Kaplan-Meyer survival plot showed decreased survival of mice when inoculated with KRT13- transduced LNCaP cells in comparison to the neo control (Figure 4G). The significance of KRT13 and cancer bone and brain metastases was confirmed in clinical prostate, breast and lung cancer specimens. Figure 4H shows positive KRT13 staining in a subpopulation of primary and metastatic human prostate, breast, and lung cancer tissues. In comparison to adenocarcinoma of the prostate, breast, and lung, stronger KRT13 staining was observed in breast and lung cancer tissues with squamous histopathology (data not included). We observed that when compared to the primary cancer specimens, KRT13 staining was more intense in all three human cancer bone metastatic specimens, and KRT13 staining was also more intense in breast and lung cancer brain metastatic specimens compared to the primary breast and lung cancer tissue specimens.

We evaluated the overall incidence of bone, brain, and soft tissue metastases in mice (N ranged from 8 to 18/group for neo-control or KRT13-transduced prostate cancer cells). Upon intracardiac inoculation of prostate cancer cells in immune-deficient SCID mice, KRT13 overexpression drove all of the tested prostate cancer cell lines toward both bone and brain, and less consistent metastases to other soft tissues, such as lymph nodes, adrenal glands, pancreas, lung, and liver. For example, LNCaP-KRT13 showed a 50% brain and 27.8% bone incidence of metastasis in comparison to indolent LNCaP cells which lacks ability to grow and metastasize in mice. In the ARCaP model, KRT13 overexpression increased tumor bone and brain metastases from 12.5 to 80% and 0 to 20%, respectively. While the aggressive 22Rv1 cells have intrinsic ability to metastasize to bone, brain and other soft tissues, KRT13 overexpression further enhanced these cells to home to bone, brain, and other soft tissues (Table 1).

**Table 1:** Increased metastatic incidence in KRT13-overexpressing cells

Site of metastasis	ARCaPE^EV^ (*n* = 8)	ARCaPE^KRT13^ (*n* = 10)	LNCaP^EV^ (*n* = 18)	LNCaP^KRT13^ (*n* = 18)	22Rv1^EV^ (*n* = 8)	22Rv1^KRT13^ (*n* = 8)
Bone	1 (12.5%)	8 (80%)	0	5 (27.8%)	3 (37.5%)	4 (50%)
Brain	0	2 (20%)	0	9 (50.0%)	5 (62.5%)	6 (75%)
Adrenal glands	2 (25.0%)	6 (60%)	0	0	7 (87.5%)	6 (75%)
Liver	0	2 (20%)	0	0	0	0
Lung	0	2 (20%)	0	0	0	1 (12.5%)
Lymph nodes	0	0	0	0	5 (62.5%)	4 (50%)
Pancreas	0	0	0	0	0	1 (12.5%)

### Gene expression profiles of KRT13- overexpressing LNCaP cells that link their preferential ability to metastasize to bone and brain

**Figure 5:**
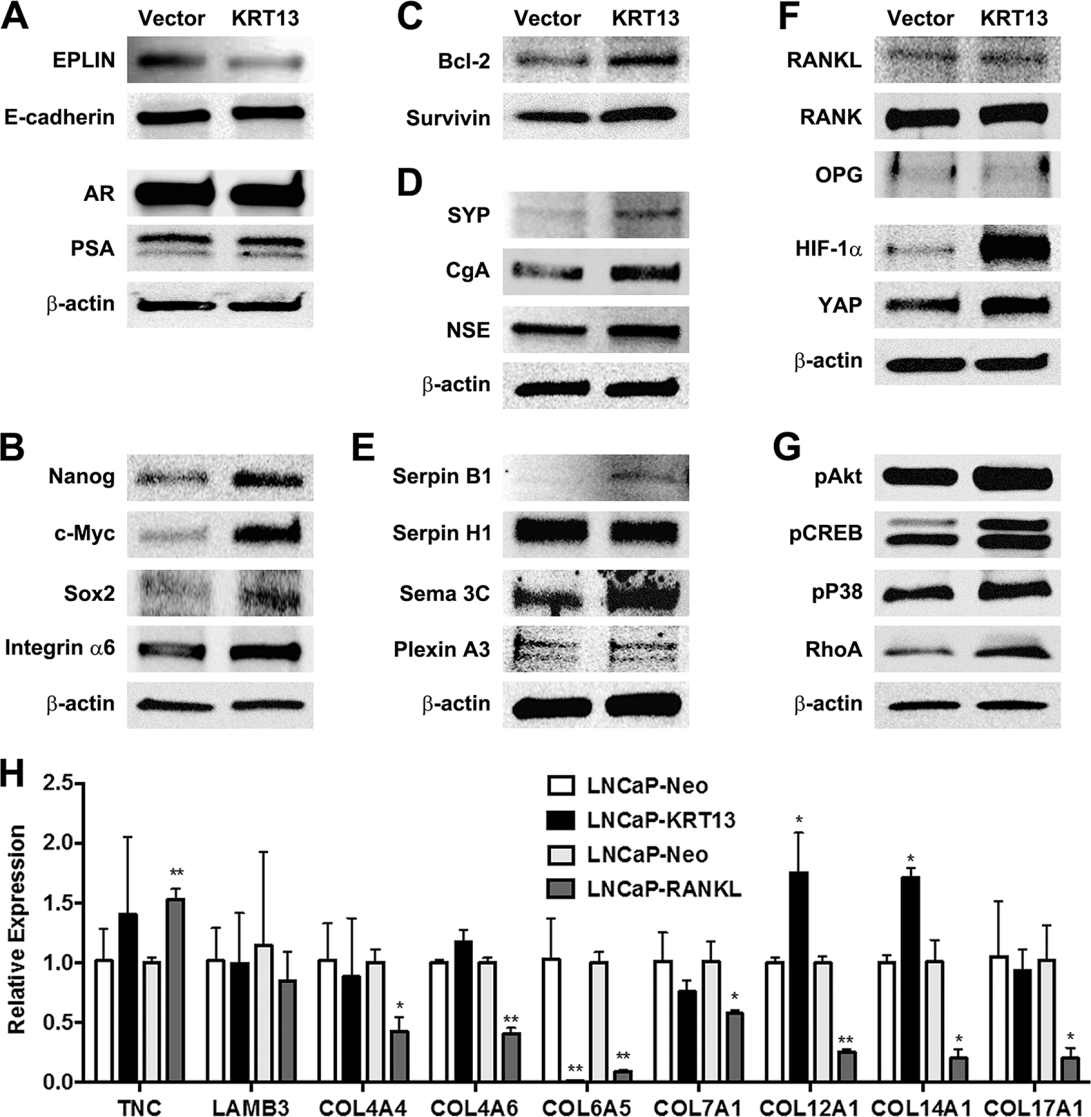
Gene expression profiles of LNCaP-KRT13 with preferential bone- and brain-metastatic potential KRT13 transduction affects expression of genes associated with EMT (**A**), stemness (**B**), cell survival (**C**), neuroendocrine (**D**), neuromimicry (**E)**, Plexin A3 level increased by 0.2- fold in LNCaP-KRT13 cells by densitometry scanning; see also Supplementary Figure S2), osteomimicry and development (**F**), and other cellular functions (**G**). Molecular size is provided in Supplementary Table S1. Differential expression of selected ECM-associated genes in LNCaP-KRT13 and LNCaP-RANKL cells analyzed by qRT-PCR (**H**). Primer sets are listed in Supplementary Table S2.

To understand the underlying molecular basis of how KRT13 reprograms prostate cancer bone and brain metastases, we adopted LNCaP as the model for more extensive gene expression profiling analyses. LNCaP-neo cells are excellent controls which provide reliable background of cells that fail to metastasize to any secondary organ sites and we have accumulated substantial background information on these models with respect to their gene expression profiles. Based on our previous success in driving human prostate cancer cells to the skeleton of host animals by overexpressing RANKL in these cells, we hypothesized that KRT13-overexpressing LNCaP cells must express similar markers associated with cell-survival-, EMT-, stem cell-, neuroendocrine cell-, developmental-, and osteomimicry-associated genes [22]. The gene expression analyses, coupled with the established metastatic profiles of tumors in mouse models, may reveal the underlying molecular biomarkers predictive of organ-specific tropism of prostate cancer cells *in vivo*. We conducted qRT-PCR and western blotting analyses of selected genes that are overexpressed in bone and brain metastatic LNCaP-KRT13 cells. Figure 5 shows a panel of pathway-associated functional genes that are preferentially expressed in human LNCaP-KRT13 cells. 1) *EMT associated genes:* In accordance with increased cell migration and invasion *in vitro*, LNCaP-KRT13 cells expressed lower level of the epithelial marker, EPLIN [27], but not the other luminal epithelial marker, E-cadherin. Despite dramatic alterations in the metastatic behaviors of LNCaP-KRT13 cells, no change was found in AR and PSA (Figure 5A). 2) *Stem cell associated genes:* Compared to indolent LNCaP-neo cells, the aggressive LNCaP-KRT13 cells expressed higher levels of genes associated with stem cell reprogramming [28, 29], such as Nanog, c-Myc, Sox2, and integrin α6 (Figure 5B). 3) *Genes associated with increased cell survival:* We observed increased expression of the cell survival genes bcl-2 and survivin in LNCaP-KRT13 cells (Figure 5C). 4) *Neuroendocrine and neuromimicry* *associated genes:* Aggressive prostate cancer cells are known to progress through increased expression of neuroendocrine and neuronal associated genes [30]. Figure 5D shows that in comparison to LNCaP-neo cells, LNCaP-KRT13 cells expressed higher levels of synaptophysin (SYP), a major synaptic vesicle protein responsible for neuronal synaptic transmission, chromogranin A (CgA) and neuron-specific enolase (NSE). In addition, we observed that LNCaP-KRT13 cells expressed selectively higher levels of some of the serpin family proteolytic enzymes, serpin B1 (but not H1), a close family gene with serpin B2, previously reported to be associated with increased breast cancer brain metastasis [31]. LNCaP-KRT13 cells also expressed higher levels of semaphorins and their plexin receptors, known to be involved in neuronal guidance function [32, 33] (Figure 5E; see also Supplementary Figure S2). 5) *Osteomimicry and developmental related genes*: Figure 5F shows that there were no alterations of RANKL, RANK, and OPG, a competitive RANKL decoy receptor, but elevated expression of developmental-related genes, HIF-1α and YAP, in LNCaP-KRT13 cells. 6) *Other cellular function associated genes:* We observed that LNCaP-KRT13 cells, when compared to LNCaP-neo cells, showed elevated phosphorylation of pAkt, pCREB, and pP38 proteins associated with increased cell proliferation; and RhoA associated with increased cell motility (Figure 5G). Because cell adhesion through interactions between extracellular matrices (ECMs) and integrins are fundamental to cancer colonization in bone and brain, we conducted a comparative survey of selective ECM genes expressed by LNCaP-KRT13/LNCaP-neo as opposed to LNCaP-RANKL/LNCaP-neo, with the latter known to have restricted metastasis to bone and soft tissues. qRT-PCR results showed altered expression of collagen subtypes in LNCaP-KRT13 cells compared to LNCaP-neo cells (see below). It is interesting to note that the patterns of the expression of ECM proteins are different between LNCaP-RANKL/LNCaP-neo when compared with LNCaP-KRT13/LNCaP-neo cells in which we observed decreased Col4A4, Col4A6, Col6A5, Col7A1, Col12A1, Col14A1, and Col17A1, in the LNCaP-RANKL/LNCaP-neo cells. In contrast, we observed elevated expression of Col12A1 and Col14A1 in LNCaP-KRT13/LNCaP-neo cells (Figure 5H). These results collectively suggest for the first time that an intermediate filament gene, when overexpressed in human prostate cancer cell lines, reprograms them toward metastasis at secondary organ sites such as bone and brain through the induction of multiple genes associated with cell survival and EMT, as well as stem cell, neuroendocrine cell, developmental, osteomimicry, and selected ECMs related to collagen family.

## DISCUSSION

Cancer metastases are potentially lethal. Models to investigate the molecular basis of cancer metastasis and screen novel therapeutics are an urgent unmet medical need. Prostate cancer frequently metastasizes to bone and soft tissues including brain [34]. While much research has focused on prostate cancer bone metastasis, increasing evidence suggests soft tissue metastases to brain, liver, lung, and adrenal glands pose greater risks of mortality and may occur at a higher frequency than previously recognized [34]. Improved molecular imaging technology detects tumor metastases more efficiently, and improved therapy for the management of castration-resistant bone metastasis enables the development of subsequent soft tissue metastases, given prolonged patient survival [35]. Interestingly, as prostate cancer progressively becomes castration-resistance, it expresses increasing levels of neuroendocrine biomarkers, EMT, stemness, therapeutic resistance, and subsequent dissemination to bone and soft tissues [36, 37]. It remains to be determined if specific biomarker expression by prostate cancer cells could predict their subsequent metastatic behaviors in mice. A model of human prostate cancer that metastasizes to both bone and brain could aid in biomarker discovery that will advance precision medicine in cancer diagnosis, prognosis, and treatment. In this study, we focused on defining the potential functional roles of KRT13 in prostate cancer progression and metastasis for four reasons. First, KRT13 is widely expressed in the cells localized in the suprabasal layer of the glandular epithelia that have been shown to contribute to carcinogenesis from cells with stem-like properties [38]. Because aggressive and therapeutic resistant prostate cancer cells often express stem cell-associated genes, we tested the possible directive effects of KRT13 in driving prostate cancer local growth, invasion and distant metastasis. Second, KRT13 was shown to be expressed in megakaryocytes localized at the sites where cancer cells could gain entry into the bone and/or soft tissue compartments (unpublished observation). We hypothesized that KRT13-positive prostate cancer cells could displace the “guarding” megakaryocytes and gain access to bone and soft tissue microenvironments. Third, KRT disruption is associated with increased actin dynamics [39] and collapsed KRT expression was observed in lung cancer brain metastatic specimens [40]. Finally, KRT14, a closely related KRT family of KRT13, was shown to be expressed in leader cells that participated in collective invasion of breast cancer metastasis [41, 42]. Thus, in this study we tested the hypothesis that KRT13 expression could alter the homing potential of prostate cancer cells to secondary organ sites. We established the following correlative observations supporting the idea that KRT13 could have a functional role in prostate cancer metastases. 1) KRT13 is expressed by developmental prostate epithelium as well as prostate cancer epithelial cells in the primary tumor and at metastatic sites (Figures 1, 2 and 4). 2) KRT13 expression in primary human prostate cancer cells predicts overall survival of prostate cancer patients (Figure 2). 3) The functions of KRT13 expression in cancers are likely cancer cell context-dependent. We observed increased KRT13 expression in some prostate cancer cell lines as they become progressively more aggressive with the potential of increasing bone-homing capability, but by contrast aggressive 22Rv1 cells capable of metastasizing to bone and brain expressed very low levels of KRT13 (Figure 3). 4) While KRT knockdown did not affect cell morphology, EMT and cancer cell invasion [43], genetically overexpressing KRT13 to levels comparable to those observed in metastatic human cancers and cultured cell lines, drove prostate cancer to undergo EMT, increased cell proliferation, migration, invasion and bone, brain and other soft tissue metastases (Figures 3 and 4). The pathophysiologic significance of these results is supported by clinical specimens in which more intense KRT13 expression was observed in human prostate cancer bone metastasis, and in human breast and lung cancer bone and brain metastases (Figure 4). 5) Gene expression analyses of KRT13-overexpressing LNCaP prostate cancer cells revealed unique expression profiles of genes associated with cell survival-, EMT-, stemness-, neuroendocrine-, neuromimicry-, osteomimicry-, ECM-, and development-related genes (Figure 5). These results collectively suggest that KRT13, previously known as a structural protein, exerts a coordinated regulation of gene expression in prostate cancer cells, driving them toward bone and brain metastases. As far as we know this is the first example, documented the tropism of prostate cancer cells to bone and brain upon overexpression of a form of KRT protein.

We reported previously that prostate cancer cells overexpressing RANKL showed enhanced expression of nine transcription factors which act as master regulatory factors responsible for the activation of over 1,640 genes associated with EMT, stem cell, neuroendocrine cell, and developmental processes [22]. We proposed a RANKL-elicited coordinated functional switch driving a subsequent metastatic cascade of prostate cancer cells to mouse skeleton and soft tissues, but not brain, through increased bone turnover by RANKL that facilitates the growth of prostate cancer cells in mouse skeleton. In the context of our previous report where we demonstrated prostate cancer bone and soft tissue metastases can be promoted by RANKL, here we show KRT13 reprograms prostate cancer bone and brain metastases occurred in a RANKL-independent manner (Figure 5F).

It has been proposed that cancer cells invading the brain often die at the brain site. In order to survive in the “hostile” brain microenvironment, cancer cells must overcome apoptotic signals and coopt other surviving cells such as endothelial lining cells in the brain by adhering to them and receiving soluble factors from the circulating microvasculature. Consistent with the ability of KRT13-expressing prostate cancer cells that develop brain metastases, we suggest the expression of neuroendocrine/neuromimicry related genes are responsible for increased communication between prostate cancer cells homing to brain and the local brain microenvironment facilitating prostate tumor growth and survival in the brain (Figure 5D and 5E). In addition to our suggestion, several other mechanisms described in the literature have been linked to the growth of prostate, breast, lung, and/or brain cancer cells in the brain through: 1) inducing EMT and survival of prostate cancer cells in a cell context-dependent manner while suppressing astrocytes, the largest population of glial cells in the brain, to prepare the brain microenvironment for the expansion of prostate cancer cells [44]; 2) secreting MMP9 and VEGF allowing the cancer cells to degrade ECMs and attract ingrowth of vascular endothelial cells [45]; 3) release of extracellular vesicles by cancer cells that trigger the disruption of blood brain barrier (BBB) via altering the actin dynamics of BBB in response to selective increase of protein and miRNA expression [46]; 4) expression of cathepsin S by both tumor cells and macrophages that could mediate transmigration of cancer cells through the BBB via proteolytic processing of the junctional adhesion molecule, JAM-B [47]; 5) antagonizing the plasmin activator (PA) defense mechanism from reactive brain stroma by producing cancer cell-associated anti-PA serpins. This protects infiltrating tumor cells in brain from apoptosis via the reduction of expression of sFasL, a death signal, and maintains tumor cell-vascular interaction, mediated by L1CAM, which provides cell adhesion and axonal guidance functions for tumor cells to coopt vascular endothelial network in the brain [31]; and 6) increased basal cell phenotype in brain metastatic lung and breast cancer cells through increased expression of Claudin [48] or FOXC1 [49].

Because there is limited information on prostate cancer brain metastasis, we reviewed our results by comparing data available from the literature on tumor growth in brain either from brain tumors or tumors metastasized to brain from lung or breast cancers, and from our own dataset of RANKL- or KRT13-expressing LNCaP cells, with the former metastasizing to the bone and soft tissues but not the brain, and the latter metastasizing to both bone and brain. These comparisons allowed us draw the following conclusions: 1) There are remarkable similarities between KRT13- and RANKL-expressing prostate cancer cells; both of these cells share common genes associated with survival-, EMT-, stemness-, neuroendocrine-, and developmental phenotypes (Figure 5). 2) There are also distinct differences of gene expression between prostate cancer cells overexpressing KRT13 and RANKL. Most notable are the overexpression of serpin B1, an anti-PA inhibitor, possibly is responsible for blocking reactive stroma defense mechanism [31], and semaphorin 3C and plexin A3, known as axon guidance ligand-receptor complexes, that possibly determine the directional movement in prostate cancer to home to brain and bone compared to bone-homing RANKL-LNCaP cells. 3) Interestingly KRT13-expressing LNCaP cells do not have elevated RANKL, RANK, and OPG and metastasize to mouse skeleton by a RANKL-independent mechanism (Figure 4). These results suggest the development of RANKL-independent inhibitors could have potential overcoming the clinical observation where the RANKL mAb, denosumab, has not been effective clinically in controlling prostate cancer metastasis and colonization in the skeleton. 4) While changes of some ECM genes are shared among KRT13- and RANKL-expressing LNCaP cells (e.g. Col6A5), two other collagen isotypes, Col12A1 and Col14A1, have opposite patterns of expression when compared between KRT13 and RANKL-overexpressing LNCaP cells. These contrasting collagen expression profiles in KRT13- and RANKL-expressing LNCaP cells could play a role in anchor prostate cancer cells in the brain microenvironment.

In summary, we established the first robust human prostate cancer bone and brain metastatic model with forced expression of KRT13 in the indolent non-metastatic LNCaP cell line. The level of KRT13 expression is equivalent to pathologic specimens of human prostate, breast, and lung cancers. Detailed gene expression profiles reveal significant resemblances between LNCaP-KRT13 and LNCaP-RANKL cells with the latter known to have restricted bone, soft tissue but not brain homing capability. Our results suggest a RANKL-independent pathway plays a key role in promoting prostate cancer bone metastasis. The bone and brain homing LNCaP-KRT13 cells were found to express serpin B1, plexin A3 and semaphorin 3C, and selective collagen-related ECMs possibly exerting anti-PA, axonal guidance, and adhesion to vascular endothelial cells to support the survival, migration, and adhesion of prostate cancer cells in the bone and brain microenvironment. At present, the mechanisms by which KRT13, an intermediate filament, could profoundly affect gene expression and cell behaviors remains unknown. We speculate that KRT13 overexpression could disturb cytoskeleton-cell junction desmosome and hemidesmosome protein complex functions, thus affecting cell adhesion and cell architecture and indirectly affecting tumor behavior, neuroendocrine phenotypes, EMT and stemness. The unanswered question is whether KRT13 functions alone by forming tonofilaments with desmosome, or co-polymerizes with other forms of keratins such as KRT4. These possibilities could be evaluated by IHC and elaborated by electron microscopy, for example plakoglobin in the intercellular adhering junctions of cancer cells, which warrants further studies [41, 42, 51].

## MATERIALS AND METHODS

### Clinical specimens

The formalin-fixed, paraffin-embedded (FFPE) tissue specimens used in this study were obtained from the following sources: 1) 24 primary, 12 bone metastatic prostate cancer tissues and one fetal prostate tissue; 20 primary, 12 bone metastatic, and 13 brain metastatic lung adenocarcinoma; and 40 primary, 8 bone metastatic, 10 brain metastatic breast cancer tissues from Sepulveda Research Corporation VA Medical Center, Los Angeles, CA, Xijing Hospital, Fourth Military Medical University, Xi'an, China, and West China Hospital, Sichuan University, Chengdu, China. 2) 44 prostate cancer tissues accrued between 1981 and 1991, generally prior to the clinical availability of PSA blood assay, were from patients who underwent transurethral resection of the prostate (TURP) primarily for relief of bladder outlet obstruction, and were subsequently diagnosed with prostate cancer. Detailed information on patient age, race, pathological Gleason score, status of distant metastasis and castration resistance, overall survival, and treatments prior to TURP for all 44 patients were provided in one of our published articles [21]. Handling of tissue specimens were conformed to the policies and practices of the respective institutions. The use of these specimens was approved by the Institutional Research Board (IRB# Pro 00021228).

### Cell culture

Human prostate cancer cell lines, CWR22Rv1 (22Rv1), PC-3, PC-3M and an epidermoid carcinoma cell line A431 were obtained from American Type Culture Collection (ATCC). LNCaP and its lineage-derived C4-2, C4-2B and isogenic ARCaPE and ARCaPBM were established and characterized in our laboratory [23, 24, 50]. The LNCaP- and ARCaP-isogenic cell lines were maintained in T-medium (Invitrogen, Carlsbad, CA) supplemented with 10% fetal bovine serum (FBS, Atlanta Biologicals; Atlanta, GA). These cell lines are authenticated for distribution by the University of Texas M.D. Anderson Cancer Center (Houston, Texas) and Novocure Laboratory (Birmingham, Alabama). A431 was maintained in DMEM (Invitrogen) supplemented with 10% FBS. The other cells were maintained routinely in RPMI-1640 (Invitrogen) supplemented with 10% FBS. All cells were maintained at 37°C in a humidified atmosphere with 5% CO_2_.

### Construction of KRT13 expression vector, lentivirus packaging and production, transduction and expression in target cells

Human KRT13 open reading frame (NM_153490) full-length cDNA was subcloned into pLVX-AcGFP1-N1 (pLV) (Clontech, Mountain View, CA) by introducing EcoRI and BamHI sites. The construct of pLVX-AcGFP1-N1-KRT13 (pLV-KRT13) was confirmed by DNA sequencing. This plasmid DNA was transfected to the 293T cells to produce lentiviral particles, following the manufacturer's instructions (System Biosciences, Inc. Mountain View, CA). Two relatively indolent (LNCaP, ARCaP**E**) and a more aggressive (22Rv1) human prostate cancer cell lines were transduced with lentivirus containing KRT13 or control vector pLV.

### Protein expression detection by immunohistochemistry (IHC,) quantum dot labeling (QDL), and western blot

The sources and dilution of the antibodies used in this study were provided in Supplementary Table S1. IHC detection of protein expression associated with tissues and cells followed our published protocols [19]. Negative controls were performed by replacing primary antibodies with species- and isotype-matched immunoglobulins prepared at the same dilution and applied to immediately adjacent tissue sections. Relative expression was scored as the intensity score times the percentage score. A quantitative quantum dot labeling (QDL) protocol was performed to assess KRT13 expression in 44 TURP tissue specimens [19]. Negative controls were included as described in IHC. Western blot analysis was performed using cultured cells reached 70–80% confluence and followed the protocols as previously described [19].

### Reverse transcription (RT)-polymerase chain reaction (PCR) and quantitative RT-PCR

Real-time PCR primer sequences were listed in Supplementary Table S2. The protocols described in our published study were followed [22].

### Cell behavioral assays

Cell migration and invasion assays were performed according to published protocols [22].

### Animal studies to assess tumor local invasion and distant metastasis

All animal procedures were performed as previously described [22] according to an approved protocol from the Institutional Animal Care and Use Committee (IACUC) of Cedars-Sinai Medical Center. Indolent parental LNCaP, ARCaPE, and 22Rv1 cells stably expressing KRT13 or control vector were transduced with firefly luciferase retroviruses (Gentarget, San Diego, CA) and selected by 200 μg/ml hygromycin (Gemini Bio Products, West Sacramento, CA). A total of 1 × 10^6^ cells in 50 μl PBS were injected via intracardiac route into 4- to 5-weeks old male C.B-17/IcrHsd-Prkdc SCID Lyst bg mice (Harlan Laboratories, Inc., Placentia, CA). The number of mice used for intratibial injection is indicated in Figure 4A. For intracardiac injection, the number of mice used was provided in Table 1. Tumor volume and metastasis were monitored and assessed weekly by bioluminescence imaging using an IVIS^®^ Spectrum or Lumina XR Optical Imaging System coupled to a data acquisition computer running Living Image software (Xenogen Corporation, Alameda, CA). Tumor first appeared at 5 to 16 weeks after intracardiac injection. Before imaging, animals were injected intraperitoneally with 15 mg/ml of luciferin potassium in PBS at 150 mg/kg body weight. A Scanco vivaCT 40 system and Bruker BioSpin 9.4T micro-MRI system were used to examine the skeleton and soft tissue lesions, respectively. Signal intensity was quantified as the sum of all detected photons per second within the region of interest. The tumor tissues were fixed and the histopathology examined.

### Statistical analysis

All assays were done in triplicate for each of the independent experiments. For 44 cases of prostate cancer tissues, the correlations between KRT13 expression and survival time, CRPC, or bone metastasis were analyzed by Log-rank test and Wilcoxon Rank Sum test, respectively. Other data were analyzed using GraphPad software (GraphPad Prism version 5.01 for Windows). Results are expressed as mean ± SEM and analyzed by a *t* test or ANOVA. A value of *p* < 0.05 was considered statistically significant.

## SUPPLEMENTARY MATERIALS FIGURES AND TABLES


