# Supplementary material for: Keratin 13 expression reprograms bone and brain metastases of human prostate cancer cells
Source: Oncotarget. 2016 Nov 7;7(51):84645–57. doi: 10.18632/oncotarget.13175 (PMC5356688; doi:10.18632/oncotarget.13175)
Supplement: Supplementary file 1 [file oncotarget-07-84645-s001.pdf]

Keratin 13 expression reprograms bone and brain metastases of human prostate cancer cells

Supplementary Materials

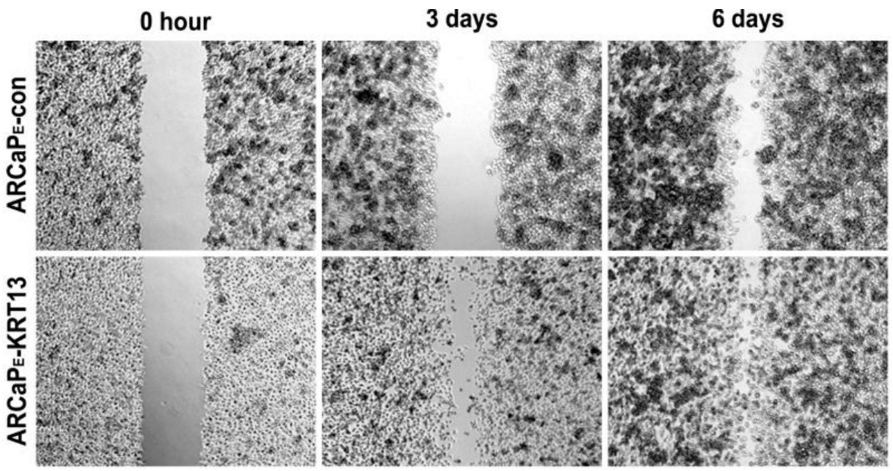

Supplementary Figure S1A: ARCaPE-KRT13 cells migrate faster than ARCaPE-neo cells as determined by wound healing assay.

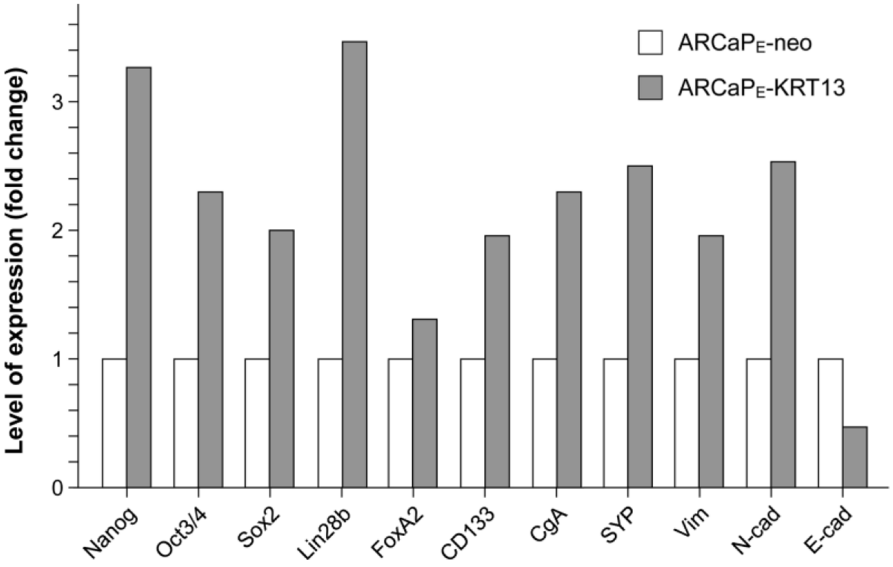

Supplementary Figure S1B: ARCaPE-KRT13 cells express genes associated with stemness (Nanog, Oct3/4, Sox 2, Lin28b, FoxA2, and CD133), neuroendocrine (CgA and SYP) and epithelial-to-mesenchymal transition (Vim, N-cad, and E-cad).

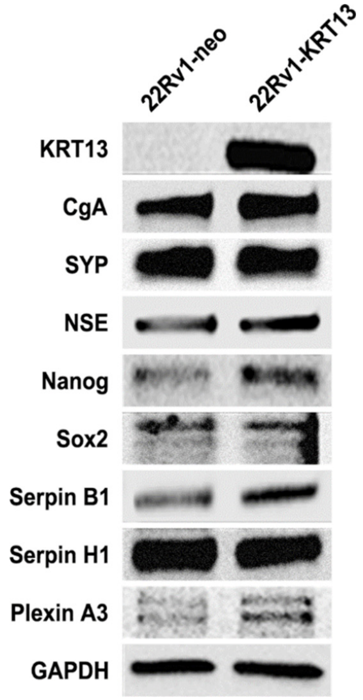

Supplementary Figure S2: KRT13-overexpressing 22Rv1 cells express higher neuroendocrine- (CgA, SYP, and NSE), stemness- (Nanog and Sox2) and neuromimicry-associated proteins (Serpin B1, Serpon H1, and Plexin A3) compared to 22Rv1-neo control cells.

Supplementary Table S1: Antibodies used in this study

Antigen	Vendor	Catalog number	Clone number	IHC/ICC dilution	Western blot Dilution	Molecular size (kD)
AR	Dr. G. Prins		PG21	1 : 200	1 : 1000	100
Bcl-2	Santa Cruz	SC-783	C21	1 : 100	1 : 500	26
c-Myc	Cell Signaling	5605	D84C12		1 : 1000	49
CgA	Santa Cruz	sc-13090	H0		1 : 500	51
KRT13	Abcam	ab92551	EPR3671	1 : 150	1 : 500	50
KRT13	Santa Cruz	sc-376047	B-12	1 : 100	1 : 500	50
E-cadherin	Cell Signaling	3195	24E10		1 : 2000	97
N-cadherin	BD Biosciences	610920	32/N-Cadherin		1 : 1000	82
Eplin	Novus Biologicals	51474-M06	1B7	1 : 500	1 : 200	92
GAPDH	Cell Signaling	2118	14C10		1 : 3000	36
HIF1a	BD Biosciences	610958	54/HIF-1α		1 : 1000	93
Integrin a6	Santa Cruz	sc-10730	H-87		1 : 500	122
Nanog	Santa Cruz	sc-134218	5A10		1 : 500	35
Nanog	Cell Signaling	4093	D73G4	1 : 200		
NSE	Santa Cruz	sc-21738	NSE-P1		1 : 500	47
Oct 3/4	Santa Cruz	sc-5279	C-10		1 : 500	39
OPG	Santa Cruz	sc-11383	H-249		1 : 500	46
pAKT	Cell Signaling	4060	D9E		1 : 1000	56
pCREB	Cell Signaling	9198	87G3		1 : 1000	35
Plexin A3	Cell Signaling	5512	D2G12		1 : 1000	208
pP38	Cell Signaling	4631	12F8		1 : 1000	42
PSA	Vector Laboratories	VP-P981	PSA 28/A4	1 : 50	1 : 500	29
RANK	Santa Cruz	sc-9072	H0		1 : 500	66
RANKL	Santa Cruz	sc-377079	G-1		1 : 500	35
RhoA	Cell Signaling	2117	67B9		1 : 1000	22
Sema 3C	Santa Cruz	sc-27796	N-20		1 : 500	85
Serpin B1	Abcam	ab181084	EPR13305(B)		1 : 1000	43
Serpin H1	Abcam	ab109117	EPR4217		1 : 1000	46
Sox2	Santa Cruz	sc-17320	Y-17		1 : 500	29
Survivin	Novus Biologicals	NB500-201		1 : 200	1 : 1000	17
Synaptophysin	Santa Cruz	sc-17750	D-4		1 : 500	34
Vimentin	Santa Cruz	sc-6260	V9		1 : 500	54
YAP	Santa Cruz	sc-15407	H-125		1 : 500	55
β-actin	Santa Cruz	sc-69879	AC-15		1 : 3000	42

Supplementary Table S2: Primer sets used in this study

Gene target	Primer sequences
KRT13	F: AGGACGCCAAGATGATTGGTT<br>R: GTGGTAACAGAGGTGCTACGG
TENASCIN	F: ACCATGCTGAGATAGATGTTCCAAA<br>R: CTTGACAGCAGAAACACCAATCC
LAMB3	F: CCAAGCCTGAGACCTACTG<br>R: GGAGTCACACTTGCAGCAT
COL4A4	F: TGAAGGGAAATCCCGGTGTG<br>R: CAGGTGGCTCTACCAACAGG
COL14A1	F: ACTCCGAGGGAAGAGAGCAA<br>R: TGTAGCAGCCACCTGTGC
COL7A1	F: CGGAACTGACCATCCAGAAT<br>R: AATAGGGTGCTCACGGTCAC
COL6A5	F: ACAGACATCTGAAGCATCTTACCT<br>R: TGACCTTGTATCTTCACCATGTTCT
COL4A6	F: CACTATGCCAGGCGCAATG<br>R: CACACACAGAGCAGCGGCT
COL12A1	F: CCTGCTAGTGGTCGTGTGCA<br>R: TCCTATTGTGGTCGTTTGCTCA
COL17A1	F: AGGAAAACTCACGTTACCCGC<br>R: AGATGCAAATTCCTTCCGAGG
Nanog	F: ATGCCTCACACGGAGACTGT<br>R: AAGTGGGTTGTTTGCCTTTG
OCT4	F: AGCAAAACCCGGAGGAGT<br>R: CCACATCGGCCTGTGTATATC
SOX2	F: TTGCTGCCTCTTTAAGACTAGGA<br>R: CTGGGGCTCAAACTTCTCTC
Lin28b	F: CTGTCAGAGCATCATGCACATG<br>R: GGGTGGCTGTGCAACATTTT
FoxA2	F: TCTTAAGAAGACGACGGCTTCAG<br>R: TTGCTCTCTCACTTGTCCTCGAT
CD133	F: CAGAGTACAACGCCAAACCA<br>R: AAATCACGATGAGGGTCAGC
CgA	F: CCCCACTGTAGTGCTGAACC<br>R: GGAGTGCTCCTGTTCTCCC
SYP	F: TGCGCTAGAGCATTCTGGG<br>R CTTAAAGCCCTGGCCCCTTCT
Vim	F: GAGAACTTTGCCGTTGAAGC<br>R: GCTTCCTGTAGGTGGCAATC
N-cad	F: ACAGTGGCCACCTACAAAGG<br>R: CCGAGATGGGGTTGATAATG
E-cad	F: TGCCCAGAAAATGAAAAAGG<br>R: GTGTATGTGGCAATGCGTTC
